# Capillary Sensor with Disposable Optrode for Diesel Fuel Quality Testing

**DOI:** 10.3390/s19091980

**Published:** 2019-04-27

**Authors:** Michal Borecki, Przemyslaw Prus, Michael L. Korwin-Pawlowski

**Affiliations:** 1 Institute of Microelectronics and Optoelectronics, Warsaw University of Technology, 00-662 Warsaw, Poland; 2 Blue Oak Inventions, 56-400 Wroclaw, Poland; pprus@boinv.com; 3 Département d’informatique et d’ingénierie, Université du Québec en Outaouais, Gatineau, QC J8X 3X7, Canada; michael.korwin-pawlowski@uqo.ca

**Keywords:** capillary sensor, diesel fuel quality, diesel fuel user, outlier data, feature vector of diesel fuel, sensor automation, artificial neural network classifier

## Abstract

Diesel fuel quality can be considered from many different points of view. Fuel producers, fuel consumers, and ecologists have their own ideas. In this paper, a sensor of diesel fuel quality type, and fuel condition that is oriented to the fuel’s consumers, is presented. The fuel quality types include premium, standard, and full bio-diesel classes. The fuel conditions include fuel fit for use and fuel degraded classes. The classes of fuel are connected with characteristics of engine operation. The presented sensor uses signal processing of an optoelectronic device monitoring fuel samples that are locally heated to the first step of boiling. Compared to previous works which consider diesel fuel quality sensing with disposable optrodes which use a more complex construction, the sensor now consists only of a capillary probe and advanced signal processing. The signal processing addresses automatic conversion of the data series to form a data pattern, estimates the measurement uncertainty, eliminates outlier data, and determines the fuel quality with an intelligent artificial neural network classifier. The sensor allows the quality classification of different unknown diesel fuel samples in less than a few minutes with the measurement costs of a single disposable capillary probe and two plugs.

## 1. Introduction

With the use of diesel fuel come considerations of its quality, cost, and type. All these factors are directly connected with the composition of the diesel fuel which consists of the fuel base, fuel improvers, and impurities. The composition of modern diesel fuel is not constant; it changes in storage due to the presence of chemically active components [1].

The type of diesel fuel may be described by the fuel base origin or the dominant technological process. Standard fuel base components may include petroleum diesel (petro-diesel), synthetic diesel (syn-diesel), fatty-acid methyl esters (FAME), and hydrogenated oils (HVO). Petro-diesel is produced in a refinery as a blend of different oils. Syn-diesel can be produced from any carbonaceous material, by gasification, purification, and conversion processes [2]. FAME is obtained from vegetable oil or animal fats with the use of transesterification reaction. HVO is a composition of alkanes obtained in the refining and hydrogenation process of vegetable oil and animal fats. Non-esterified vegetable oil was also considered as fuel a bio-component [3].

Modern petro-diesel fuel is composed of about 74% saturated hydrocarbons—primarily alkanes, 25% aromatic and acyclic unsaturated hydrocarbons which are chemically active and 1% of impurities and additives. The bio-diesel fuel, beside petro-diesel components, has to contain FAME or HVO components. The FAME component concentration in diesel fuel is limited, as FAME is a chemically active component.

The diesel fuel quality is an important and complex issue [4,5]. It may be considered from different points of view. Fuel producers, fuel traders, fuel users, ecological organizations, and public health points of view and economic interests may differ [6,7,8,9].

On the diesel fuel user’s side, low cost and high quality of the fuel sold at fuel stations are important [10]. The quality of diesel fuel impacts diesel engine operating characteristics, such as starting ease, lack of engine stalling at low speeds, sufficient power, low-temperature operability, low engine noise, and low engine wear [11]. The set of parameters including starting ease, engine stalling at low speeds, and sufficient power may be associated with fuel ignition quality described by fuel producers as the cetane number [12,13]. Low-temperature operability of diesel fuel is important in winter conditions, and it directly depends on the main fuel component’s composition and concentration of FAME [14]. Low engine noise may be connected with fuel lubricity [15]. 

On the fuel traders’ side, fuel storage stability and fuel quality are important. The fuel quality is here understood as consumer satisfaction, while fuel storage stability still impacts the actual fuel quality [16]. On the fuel producer’s side, the quality of diesel fuel is determined by quality standards. These quality standards differ for petro-diesel and bio-diesel fuels. The most popular standards of petro-diesel fuels are ASTM D 975 introduced by the American Society for Testing and Materials and EN 590 introduced by the European Committee for Standardization [17]. Respectively, the standards for bio-diesel fuels are the ASTM D 6751 in the USA and the EN 14, 214 in Europe. In this approach, the quality of fuel can be defined as a coincidence of a set of laboratories measured fuel parameters with defined standards ranges. It should be noted that the mentioned set of parameters is extensive and measurement methods are expensive and time-consuming. For example, the basic parameters set includes cetane number, density, viscosity, the fractional composition of distillation, and induction time as well as bio component contents in fuel composition. The accurate measurement of the basic fuel quality parameter, the cetane number, requires examination according to American Society for Testing and Materials (ASTM) standard D 613. It involves burning of fuel at a constant speed in a rare diesel engine called a Cooperative Fuel Research (CFR) engine for 47 minutes. Moreover, the mentioned fuel parameters ranges are the result of different administration regulations adopted by different countries [18]. Therefore, a formal exploration of diesel fuel quality is a complex task. Fortunately, in the EU, according to Directive 98/70/EC and 2003/17/EC on petrol and diesel fuels quality, all the Member States are committed to scrutinizing the quality of fuels sold at the filling stations in their respective countries [19]. 

Novel sensing concepts of diesel fuel quality propositions base on infrared spectroscopy [20,21] and dielectric spectroscopy [22,23] are mainly oriented to petro-diesel/bio-diesel blends’ content assessment. It is worth noting that infrared spectroscopy measurement results may cover the concentration composition of hydrocarbons in diesel fuel, but the identification method of fuel quality that is based on a measured profile of hydrocarbon concentrations, is still under investigations. Fluorescence sensing methods are also under investigations. Ultraviolet excitation and emission analysis may lead to the classification of fuel type [24], indications of fuel degradation stage [25], and fuel stability [26]. 

Measurements of a bio-component presence in diesel fuel may be performed with the use of viscometer [27]. Furthermore, the density measured at a range of temperatures can be used to provide a concentration percentage of binary solutions [28]. Therefore, some sensor producers assume that diesel fuel is a binary solution of petro- and bio-components and propose sensors for diesel fuel measurements accordingly [29]. In fact, diesel fuel is not a binary solution; the petro-component includes a significant number of different subcomponents. 

The multiparametric sensing concept leads to a commercially valid proposition of fuel sensors where viscosity, dielectric constant, temperature, and density are measured at the same time and are used for fuel type classification [30]. However, it should be noted that some sensor producers postulate that their products, working on the mentioned rule, are oriented to measure fuel quality, while these products are mainly adapted to sense FAME concentration in fuel. Against that background, the examination of the capillary action, that introduces viscosity, density, and surface tension information to fuel type classification, enables simplified fuel fit-for-use inspection [31]. 

New propositions of sensors oriented at diesel fuel parameters are under investigation, as, for example, an electronic nose for the discrimination of weathered petroleum products [32]. However, diesel fuel quality, in simplification, can be considered as ignition quality and low volatility at standard environment temperatures [33]. The ignition quality can be characterized by spray formation parameters during fuel injection in the engine chamber [34]. The spray formation, considered as a fuel parameter, depends mainly, at a selected temperature, on viscosity, surface tension, low boiling temperature, and volatility of fuel [35]. However, the spray formation, considered on the engine hardware side, depends on injection pressure, injection vessel diameter, and chamber parameters [36,37]. Meanwhile, the core findings of diesel fuel quality sensor development that is based on a capillary optrode are characterized by a set of interesting features:

- A capillary sensor with a principle of operation close to the measurement of fuel injection parameters enables time of examinations below a few minutes [38].
- Diesel fuel examination with local sample heating, which is positioned in a smart photonic capillary, show the possibility of precise fuel type classification [39]. The biggest drawbacks of such capillary sensors are complications in the technology of the capillary optrode preparation and positioning in the head bed.
- Capillary sensor operation with a disposable optrode sometimes generates outlier data [40] as a result of improper measurements.
- Capillary sensors with forced local sample heating are fit to automatically collect the characteristics of signal features [41].

Thus, the main aim of this work is the proposition of a capillary sensor that is oriented to the fuel user and the fuel trader, providing information on the fuel quality in less than few minutes with reasonable costs of the examination.

## 2. The Idea of the Head of the Capillary Sensor with Disposable Optrode for Diesel Fuel Quality Examination 

**Figure 1:**
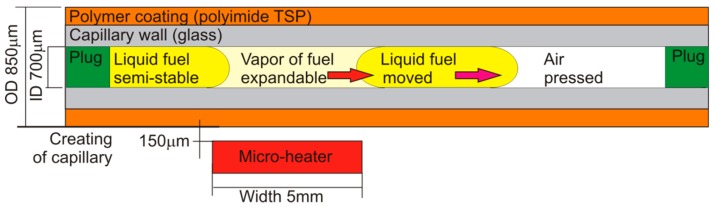
Diesel fuel vapor phase creation and expansion in one defined direction as a result of local sample heating.

The sensor head idea was inspired by one of the most critical elements of the diesel engine from fuel quality point of view; that is the fuel injector. Modern direct injection diesel engines operate through the injection of liquid diesel fuel into the engine combustion chamber. The liquid fuel is subjected to large pressure and temperature gradients inside the injector and the nozzle. The actual dimensions of the typical nozzle diameters can vary from 50 to 200 µm [42]. The character of the fuel flow through the nozzle depends strongly on the temperature and pressure difference. The input pressure can vary from 15 MPa to 110 MPa, while the output pressure is of the order of 6 MPa. Typical temperatures inside the fuel injection nozzle are from 235 to 275 °C. Therefore, fuel passage is often associated with local boiling of the fuel that results in local creation of bubbles of fuel vapor. The flow can be described as a gas and liquid phase spray in which thermodynamic transformation occurs. The fuel enters the cylinder in about a few milliseconds [43]. After the injection of fuel into the cylinder, the spray vaporizes and inflames. The quality of fuel ignition is often correlated to spray formation [44]. The low-quality fuel is injected in the form of a stream or gas phase. The flame temperatures in the cylinders are about 1500 °C, and the wall temperatures are under 350 °C. Therefore, the direct replication of the mentioned phenomena occurring in the nozzle in a small portable sensor is very difficult. On the other hand, while the thesis that heated to boiling point fuel sample flow characteristics in a vessel similar to the injector can be used for fuel characterization seems right. The practical application of the sensor requires a simplification of the previously presented optrode construction [39]. The simplest vessel that can be used for monitoring of forced by temperature and pressure fuel flow is a capillary. When the capillary is partially filled with a fuel sample and closed from both ends, locally increased temperature of the diesel fuel sample results in vapor phase creation and expansion in one defined direction, as presented in Figure 1. It should be noted, that repeatable local heating of the diesel fuel sample to the low boiling point can be realized with a specific and properly positioned micro-heater [45]. The capillary used in experiments is a Polymicro TSP700850. A thickness of the capillary glass wall is about 50 µm (±15 µm), while the thickness of the polyimide coating is 24 µm. It should be noted also, that capillary coating is made of standard type of polyimide (TSP) with thermal stability that reaches up to 350 °C, and whose capillary coating is transparent optically at 780 nm.

**Figure 2:**
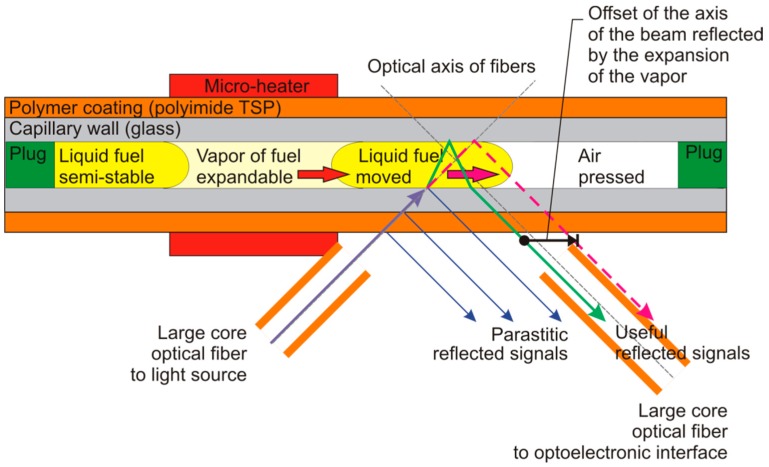
Diesel fuel vapor phase creation and expansion monitoring.

The fuel heating to low boiling point generates a vapor phase of the fuel with parameters characterized by fuel vapor phase pressure at the specified temperature. The fluid flow type may be turbid or laminar. It depends on viscosity, the surface tension of the liquid phase as well as of the gas phase vapor pressure. It is worth noting that these parameters are used to model spray formation during fuel injection to the ignition chamber. The liquid and gas phase movement can be achieved with the use of optical fibers. The point for observation is located in the capillary on the right side of the micro-heater. The position of the capillary is presented in Figure 1. The observation of the capillary from one side is based on the refractive index differences between gas and liquid phases. The simplified scheme of the important, from sensing point of view, light beam paths in the capillary head is presented in Figure 2. The main simplification is the two-dimensional presentation of the cylindrical structure of capillary; the minor simplification is the assumption of the low thickness of capillary wall comparing to the diameter of the capillary hole.

The light beam from a large core fiber lights the optrode’s outer wall at 45 degrees. In this wall, the first light beam fragmentation happens due to Fresnel reflection. Next, similar beam fragmentations occur at the contact of different optrode wall materials—the polymer coating and glass wall as well as in the capillary wall and inner substance contact. These beams form parasitic signals. The main optical beam enters the capillary hole. When the hole is filled with the liquid fuel sample, significant refraction of the light beam occurs, as shown by the green line on Figure 2. A similar situation occurs for the light beam that leaves the capillary hole. When the capillary hole is filled with the gas phase of fuel, such refraction of light, in the proposed optical configuration, is too small to direct signal into detection fiber. The difference in refractive indexes of the gas and liquid phase results in an offset of reflected beams that are directed to the head of the large core optical fiber which is connected to the optoelectronic interface.

Converting of quite a complex sensing idea into a working sensor requires a structured development methodology. Obtained intermediate results of the development are grouped together in Section 3. As the subject of the sensor is diesel fuel quality testing, first, the diesel fuels to be used in experiments have been selected and tested which is presented in Section 3.1. Details of capillary sensor construction and the basic principle of operation are presented in Section 3.2. Necessary initial data processing is presented in Section 3.3. As the developed sensor is person operated, the principles of outlier data generation and rejection are presented in Section 3.4. The diesel fuels classification with the use of artificial neural network assumptions and results are presented in Section 3.5. Obtained results referred to accessible, on the internet, data discussion announced in Section 4. The conclusions are gathered in Section 5.

## 3. Capillary Sensor with Disposable Optrode Development

### 3.1. Diesel Fuels for Experiments

The commercial diesel fuels samples of different qualities were subject to examination with the developed sensor. We defined the fuel quality types as premium, good, and conditionally acceptable. As the premium quality fuel, we evaluated the clear and fresh petro-diesel that met EU standards. The standard quality fuel was a petro-diesel mixture with seven bio-components. The conditionally acceptable fuel base consisted of 100% FAME. The fuel condition can be good when the fuel is fresh or bad when the fuel is out of date or damaged. In the analyzed case, bad fuel consisted of the conditionally acceptable fuel that was degraded by storage for three years in a standard polymer container filled to 50% of its volume. The parameters of the fresh fuels according to EU standards are presented in Table 1. The damaged fuel was characterized by the presence of sediment; therefore, it did not meet the EU standards. However, diesel fuel, even under standard storage conditions, is subject to sedimentation. Therefore, the fuel samples, before filling of a capillary, were shaken in a standard container and then mixed in a laboratory vessel.

### 3.2. Capillary Sensor Construction and Basic Principle of Operation

**Figure 3:**
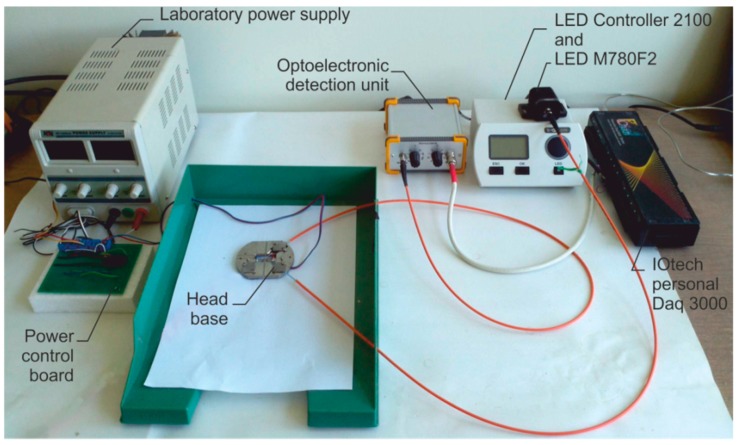
Examined sensor view, based on [40].

The examination described in this paper was performed with the hardware set-up presented in [40]. The sensor set-up view is presented in Figure 3. It is a laboratory set-up consisting of in-house-made components as the head base, the power control board, optoelectronic interface and of commercial modules of a basic laboratory power supply, fiber coupled light emitting diode (LED), high power LED controller, acquisition card, and a personal computer with software. As the light source, the LED Controller 2100 and LED M780F2 were used. The nominal wavelength of applied LED is 780 nm. To improve the rejection of ambient light influence on the experimental results we used light electrically modulated with 1 kHz frequency. The optoelectronic detection unit is of our own construction in which the S8745-01 (integrated optical sensor consisting of Si photodiode, operational amplifier, feedback resistance, and capacitance), AD8253 (instrumentation amplifier with digitally programmable gains), UAF42 (universal configurable active filter), AD536 (true root mean square to direct current converter) and AD8250 (instrumentation amplifier with digitally programmable gain) are the key components. The opto-electronic interface had a sub-miniature version A (SMA) fiber input. The sensitivity of the optoelectronic module is 2 mV/nW. Limited by electromagnetic noise the proposed setup enables to measure signals with 0.01 s time resolution, in the range from 10 nW up to 500 nW with 2 nW accuracy. The optoelectronic interface was connected to a personal computer through an analog input IOtech personal Daq 3000 16 bit/1 MHz USB data acquisition system [46]. We fed the micro-heater from a laboratory power supply by an in-house-made relay board controlled by the digital output from Daq. 

**Figure 4:**
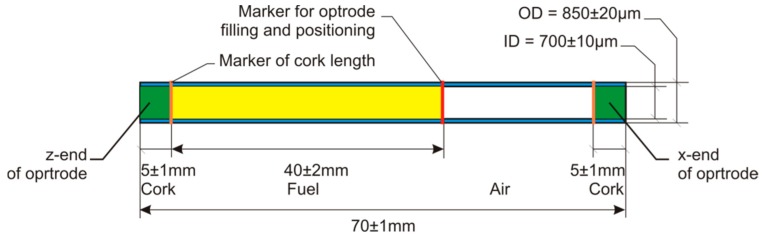
Capillary optrode properly prepared for fuel examination, based on [40].

The sensor’s head consists of two separate main elements: the base and the disposable capillary optrode. The micro-heater and optical fibers are integrated with the base. The base is also used to position the optrode. The schematic drawing of the optrode used in this work is presented in Figure 4. The optrode capillary is a Polymicro TSP700850 with printed three red markers. The most important marker, placed in the middle part of the capillary, is used as a pointer of fuel filling range and as a pointer of optrode position in the head base. The two other markers are used as pointers of the proper length of insertion of elastic polymer corks. During experiments, the capillary optrode was filled by the open z-end, to the central marker with capillary force use. Then, the x-end of optrode, which was in the air, was closed by a finger. The z-end of capillary was removed from a vessel with a sample and was pushed into a polymeric mass layer of 5 mm height positioned in the container. Then the x-end of the capillary was closed in the same way. Next, the capillary was positioned in the head base. The measurement cycle was started with running the software script.

**Figure 5:**
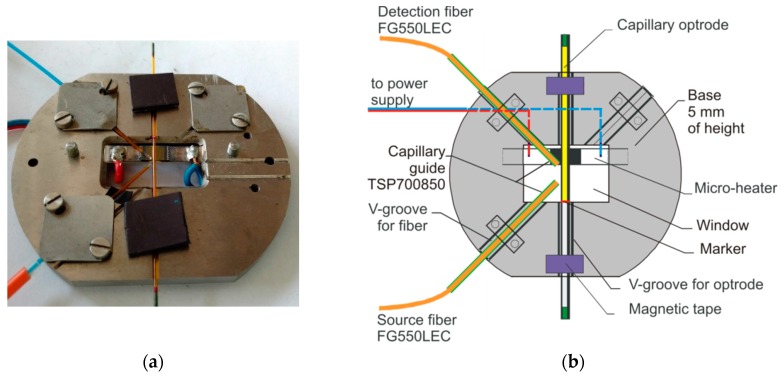
The head base with optrode: (**a**) View; (**b**) Scheme.

The schematic drawing of the sensor head used in this work is presented in Figure 5. The base of the sensor head is made of 5 mm height alumina roller. In this roller, v-grooves used for optical elements adjustment were milled. The top and bottom of the roller were cut off to simplify capillary optrode positioning. In the middle of the base a window was cut off. This way the micro-heater mounted to the bottom of the base could locally heat the optrode. The micro-heater is a hybrid construction of a monolithic silicon carbide heating element positioned on the plate made of ceramics. This ceramic plate is partially covered with metal contacts pads that are covered with thin films of BaTiO_3_ [47]. An additional function of the ceramic plate connected to the head base is cooling of the electrical connection. The lower edge of the window in the head base was used to positioning the capillary optrode.

The step-index multimode fiber optic patch cable with SMA connectors M37L02 from Thorlabs cut in half was used to prepare two probes. This patch cable was made using FG550LEC fiber with the outer coating diameter of 630 µm, while buffer outer diameter was 1040 µm. The ends of fibers, with the buffer removed, were inserted into 2 cm sections of TSP700850 capillary to allow matching the planes of the optical axes. The optical axes cross point was in the middle of capillary optrode. The optical fibers were positioned as close to the capillary optrode as possible. Thus, the light beams of the signals crossed and reflected at the filled optrode, and entered the detection fiber. When the capillary was filled with gas, the mentioned signal beam offset resulted in proper decoupling of the beam from the detection fiber, as presented in Figure 2. The parasitic reflected signals presented in Figure 2 were not coupled to the detection fiber in the head. In the described construction of the sensor the optical signal average power reflected on an empty capillary was at a level of 0.25 V, while the signal for a capillary filled with fuel was at the level of 4.5 V. The variation in LED intensity over time and capillary optrode imperfections were corrected with applied calibration procedure that acts with measured initial values. The calibration principle led, the measured value of the initial point, to fit in the range from 4.0 V to 4.5 V.

The sensor provided information that was represented by the dynamic changes of the signal during local heating and cooling of the sample in the forced measurement cycle. In this work, the micro-heater power was set to 10 W. The measuring procedure was started with switching on the micro-hearer power and registration of signal. The micro-heater was automatically switched off when the amplitude of the monitored signal crossed from above 2.0 V. 

**Figure 6:**
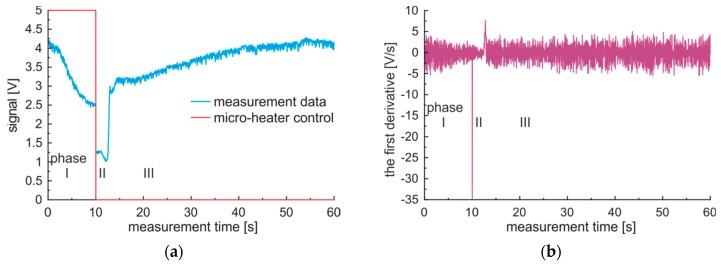
Sample signals of the forced measurement cycle obtained for premium diesel fuel: (**a**) Signal; (**b**) The first derivative.

The raw data, micro-heater control signal, and calculated first derivatives for a sample of premium diesel fuel in a measurement cycle collected with 100 Hz acquisition frequency are presented in Figure 6a. The first derivative calculated with the five point’s method, presented in Figure 6b. The forced measurement cycle contains, according to Figure 6, three phases: I—local sample heating up to observed gas phase creation, II—gas phase movement, cooling and reabsorption, III—liquid sample cooling.

The simplest description of these situations may be as follow. The first phase is represented by the optical signal’s slow decrease as the refractive index of oils decreases as a function of the temperature [48]. The second phase is represented by a sudden depression of the signal as the gas vapor expanded from the micro-heater area is present in the optically monitored area for some time. Then, the gas phase is cooled by the surrounding environment and reabsorbed by liquid fuel. The third phase is represented by a slow signal increase as the liquid fuel is cooled down. It is worth noting, that the fuel parameters are correlated with these phases and phases’ transition. Specific heat and lower boiling point are correlated with the duration of the first phase. The heat of vaporization, vapor pressure, and viscosity are linked with the transition speed from phase I to II. The heat of vaporization, vapor pressure, and liquid fuel bubble parameters as surface tension as well as viscosity are associated with the duration of the second phase. The signal damping is correlated with fuel lubricity, as for monitored gas phase presence a residual fuel remains on inner capillary walls. The signal in phase III may indicate a turbid or laminar flow of fluid in the capillary. The turbid flow results in a low-level signal as mixed air bubbles are not absorbed in the liquid fuel.

### 3.3. Initial Data Processing

The detection of signal levels and measurement phases’ transitions are not perfectly accurate due to the presence of noise in the signal and the first derivative. The noise can be cleaned with the use of exponential filtering of raw data signals, which has an influence on analyzed signal and the first derivative. Data analysis results in the observation of a minimal reduction of signal noises for the damping factor set to 0.2, while the 4.0 V/s noises of the first derivative are observable for damping factor set to 0.5 and 2.5 V/s for damping factor set to 0.8. The implementation of exponential filtering with 0.8 damping factor of measured data of premium fuel, results in the ratio of the value of the second peak of the first derivative versus noise increases to 3.0 from 1.5 observed for not filtered data. Thus, the detection of phase II and the transition from phase II to III with exponential filtering becomes more reliable.

**Figure 7:**
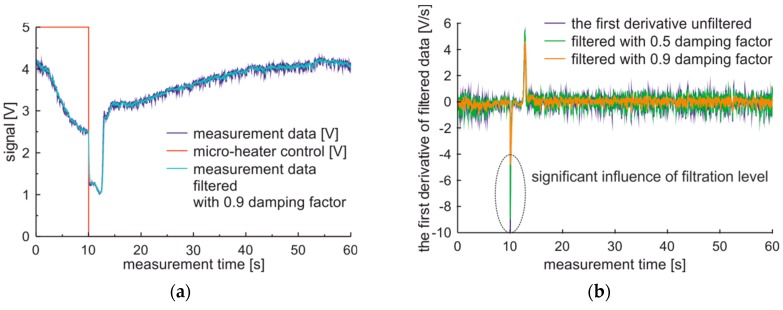
Measurement cycle obtained for premium-diesel fuel and results of the first derivative filtration: (**a**) Signals raw and filtered; (**b**) The first derivative of filtered signal.

The first derivative also can be filtered. The characteristics of the first derivative of premium diesel fuel data filtered with damping factor set to 0.9, calculated with the five points method, and processed with a different level of filtration are presented in Figure 7. The influence of the filtration level on the first derivative is visible. The noise level and peak values of the derivative decrease with the increase of the filtration level. It can be observed, that use of filtration with the damping factor set to 0.9 can be positive in the case of visualization of measurement data, while filtration with such damping factor used on of the first derivative can have negative influences on measuring rapid signal changes.

**Figure 8:**
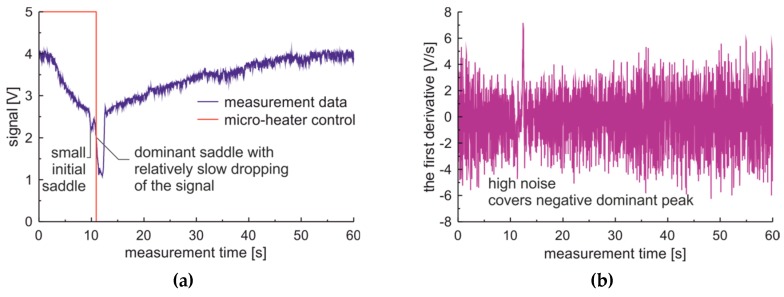
Signals and the first derivative of the measurement cycle obtained for 100% bio-diesel fuel for the sensor located in an area with saturated WiFi transmission: (**a**) Raw signal; (**b**) The first derivative of the raw signal.

The measurement data of 100% bio-diesel fuel, prepared on a FAME base, obtained from the sensor located in an area of saturated WiFi communications are presented in Figure 8a. The measurement data show the presence of two saddles. The first initial saddle is small. The dominant saddle is characterized by a relatively slow drop in the signal.

**Figure 9:**
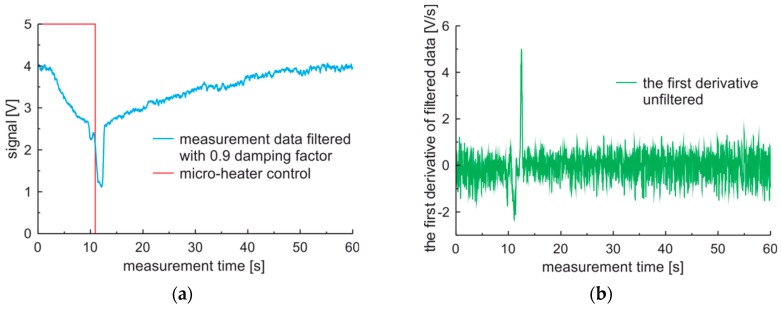
Filtered signals of the measurement cycle obtained for 100% bio-diesel fuel for the sensor located in an area with saturated WiFi transmission: (**a**) Filtered signal; (**b**) The first derivative of the filtered signal.

Significantly, the electromagnetic noise inducted by WiFi results in a high level of noise presented in the first derivative of the raw signal as presented in Figure 8b. This level of noise and the measurement data of bio-diesel fuel prepared from FAME made the first derivative of raw data useless, as noise covers the negative peak of the derivative at the dominant saddle. For such case results of the heavy filtration of the signal with the use of exponential filtering with the damping factor of the signal set to 0.9 are presented in Figure 9. In the presented case, the unfiltered first derivative of filtered signal can be applied to determine the slope of the dominant saddle. It is worth noting that the orders of dominant peak’s absolute values are different for premium diesel and for bio-diesel based on FAME. The conclusion of the presented analysis is that measured raw data for further analysis will be filtered with a damping factor of 0.9 while the first derivative will be calculated with the five point’s method.

### 3.4. Outlier Data Rejection 

The evaluated capillary sensor is not equipped with automatic optrode filling and positioning utilities. Therefore, an untrained operator may introduce an improperly prepared optrode to the examination or perform examinations with the optrode improperly positioned in the base. Such procedure imprecisions result with outlier data generations. 

**Figure 10:**
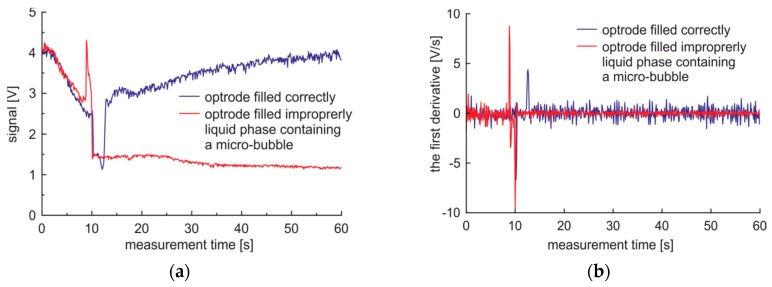
Measurement series of premium fuel for optrode properly filled and for optrode filled with gas phase introduced to fuel sample from cork side: (**a**) Signal; (**b**) The first derivative.

The example of measurement series and their first derivatives for the optrode properly filled and for the optrode filled with gas phase introduced to the fuel sample from the cork side are presented in Figure 10. Unwanted air phase forms a micro-bubble. This micro-bubble moves during local heating of the optrode. This movement results in the peak of the signal that appears before the local transition from liquid to gas phase of the fuel sample. This data can be optically classified as an outlier. The first peak of the signal is formed in the opposite direction to the typical initial signal of the saddle. Therefore, the sign of the first peak of the first derivative can be used as an outlier data pointer.

**Figure 11:**
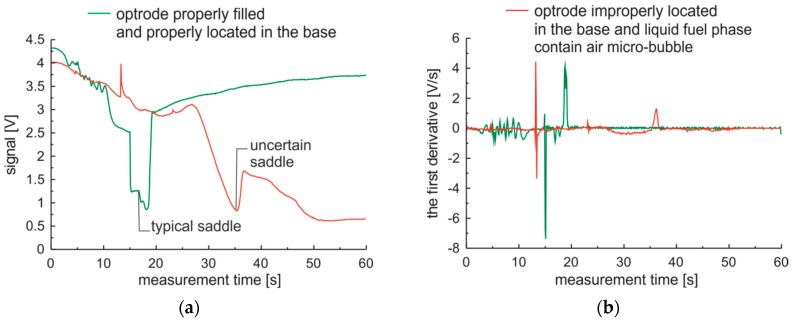
Measurement series of standard fuel for optrode properly prepared for examination and for optrode improperly filled and improperly located in the base: (**a**) Signal; (**b**) The first derivative.

Examples of measurement series and their first derivatives for the optrode properly filled, in comparison with the optrode filled with gas phase and for the optrode improperly positioned in the base are presented in Figure 11. The unwanted air phase forms a micro-bubble as in the previous case. The improperly positioned optrode there is represented by the optrode of which one side was elevated by an additional 100 µm compared with the proper position. The outlier data generated by micro-bubble of air look similar to the previous case presented in Figure 10.

The typical saddle of proper measurement differs in shape and time position from that for the improperly positioned optrode. The saddle is present, but the speed of its developing is somehow slow. The peak with a negative sign is fuzzy.

**Figure 12:**
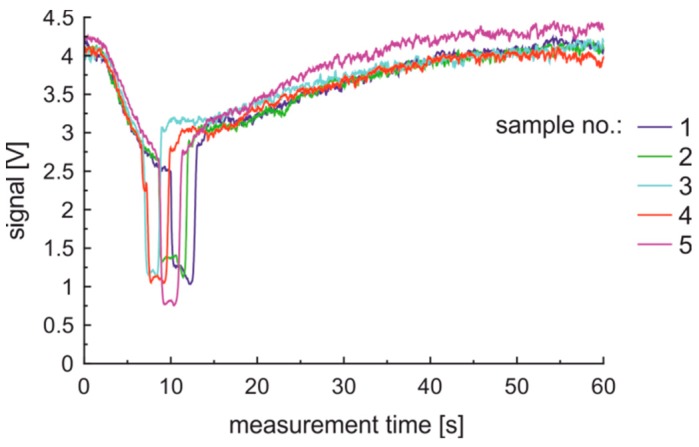
Data collected for five samples of the same premium diesel fuel.

Basing on the obtained results the first derivative of properly measured data is characterized with the first dominant peak of negative sign and the second dominant peak of positive sign. Outlier data classification can be based on the presence of two dominant peaks of the first derivative where the first peak is positive and the second negative. Uncertain data classification can be based on the absence of the dominant negative peak of the first derivative; instead of the peak a valley may be present. In the following examination of fuels classification the outlier and uncertain data were rejected using the mentioned method. Data from random selected five samples of the same premium diesel fuel are presented in Figure 12. 

This data shows that the course of the measured time series has the same character. However, the classical data analysis with the use of time window should require a basic method adaptation. In this paper, we have reached 95% of correct data, as a result of accurate laboratory work.

### 3.5. Fuels Classification with the Use of Artificial Neural Network

**Figure 13:**
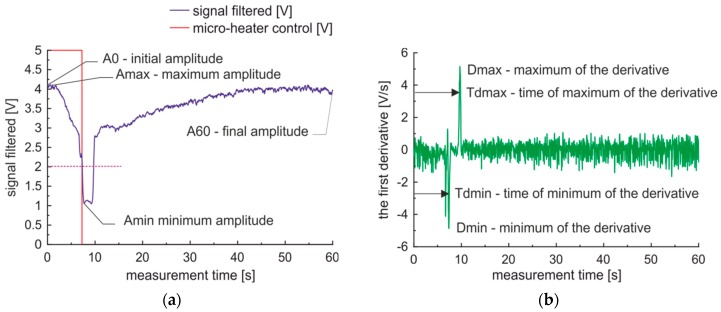
Data used to generate input vectors for fuel classification: (**a**) Signal; (**b**) The first derivative.

The fuels classification was done with Qnet software oriented to the development of artificial neural network (ANN) and working with multilayer perceptron as a classifier. We examined two cases. The first case refers to the output of ANN set to inform of fuel quality type. The second case refers to the output of ANN set to fuel condition. Our numerical experiments indicate that multilayer perceptron with seven inputs, one hidden layer with five neurons and an output layer with one neuron, all equipped with sigmoid transfer functions performs the classification task better than more complex or simpler artificial neural networks. In both cases of the ANN experiment, the input data parameters presented in Figure 13 were processed to the form presented in Table 2.

The ANN experiment input data consist of 45 vectors collected for fuels samples presented in Table 2. The ANN classifiers tests were prepared with premium, fresh fuel samples that came from a different origin than samples used for ANN learning.

**Figure 14:**
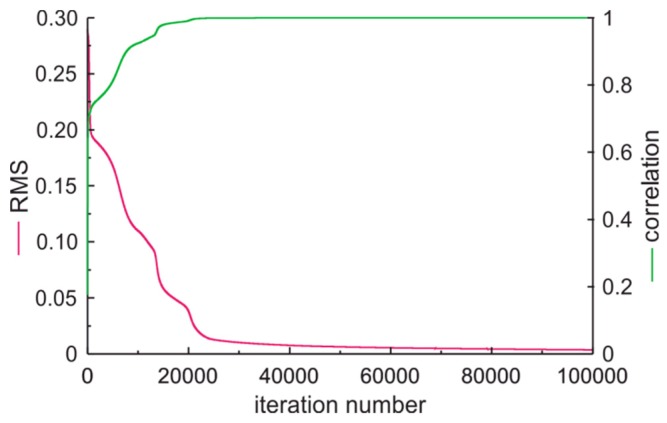
The artificial neural network (ANN) learning process of fuel quality type classification.

**Figure 15:**
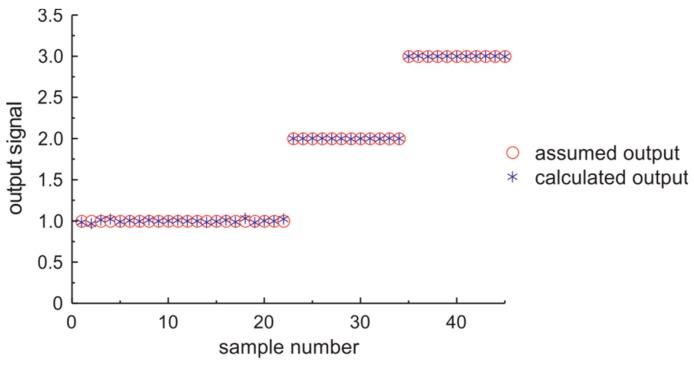
The assumed output values versus calculated output values of ANN classifier of fuel quality type.

In the first case the output of ANN was assumed as 1—for conditionally acceptable fuel, 2—for standard fuel, and 3—for premium fuel. The ANN learning process is illustrated in Figure 14. The assumed output values versus calculated output values of ANN classifier of fuel quality type are presented in Figure 15. These data confirm the proper preparation of vectors passed to ANN and of proper ANN selection. 

**Figure 16:**
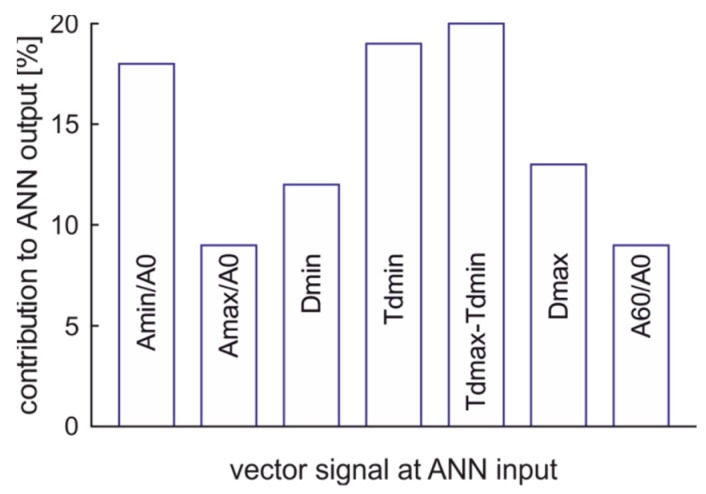
The contribution of input values to ANN classifier output of fuel quality type.

The contribution of inputs to the output of ANN classifier is presented in Figure 16. These data confirm the complex structure of the sensor. All input vector signals contribute to the classifier output. The most important contributions are the time of vapor phase creation, time of vapor phase presence, and the minimum of the signal. This minimum is linked with lack of thin films of fuel that remain of the capillary’s inner walls during vapor phase presence.

The ANN classifiers output of fuel quality type tested with premium, fresh fuel samples that were from a different origin than samples used for ANN learning, shows the proper classification of eight samples and one missing. In our opinion, such classification results of unknown samples are quite satisfactory, as the learning set is not big compared to the testing set. 

**Figure 17:**
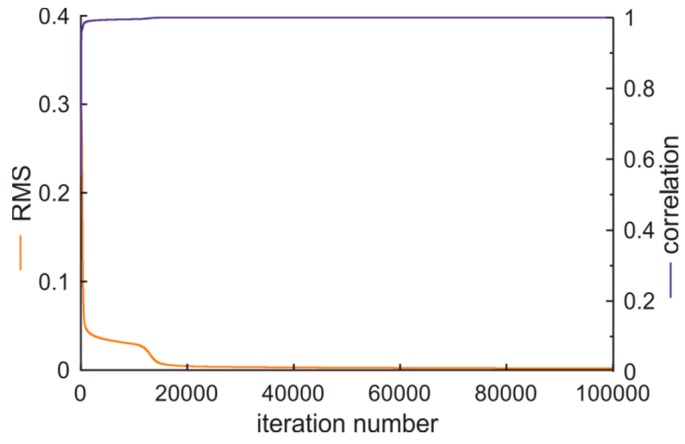
The ANN learning process of fuel state classification.

**Figure 18:**
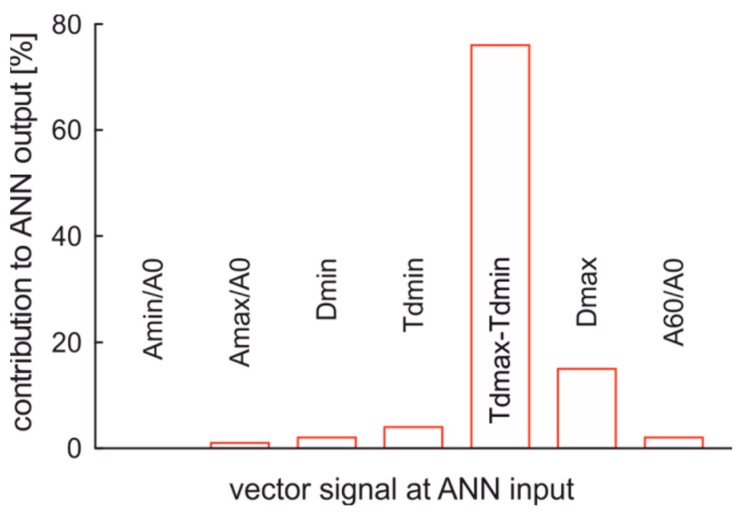
The contribution of input values to ANN classifier output of fuel state.

In the second case, the output of ANN was assumed as 1—for good fuel state and 0—for a damaged state. The ANN learning process is illustrated in Figure 17. The learning process was, in that case, faster, and results were more precise than in the previous case. The assumed output values versus calculated output values of ANN classifier of fuel state were almost the same when presented on the chart. The contribution of inputs to the output of classifier of fuel state is presented in Figure 18.

In the analyzed case the most important contribution is the time of vapor phase presence. That validates the construction and the principle of work of the sensor, which bases on local vapor phase creation.

In addition, this classifier of fuel state has been tested with premium, fresh fuel samples that are from a different origin that samples used for ANN learning. The results show the proper classification of all nine samples.

## 4. Results Discussion

It is interesting that diesel fuel level sensors are the main answer in commercial sensors’ domain for an internet query with “diesel fuel sensor”. There are set of sensors suitable for diesel fuel level measurement, for example, PT124B-224 is a capacitive sensor, QTYB QT is a radar level meter, GUT810 is an ultrasonic sensor, and HL200306 is a mechanical sensor. The first item that appears after query “diesel fuel quality sensor” is the LH-BW-180807-16 sensor used to AdBlue presence indicator. There are a few commercial sensors that can be oriented to measure, in-situ, selected fuel parameter as, for example, density or viscosity.

The commercial sensor MEAS FPS 2800 and fuel quality sensor from SCI enable multiparametric measurement of density, viscosity, dielectric constant, temperature of sample, and level or water interface of fuel, [30]. The density, viscosity, and dielectric constant may lead to liquid fuel type classification as gasoline, petro-diesel, and biodiesel or jet fuel. This sensor also may point the petro-diesel fuel contamination with water, urea, FAME, glycerol, and methanol. The presented results are in agreement with data accessible in [49] where sensor MEAS FPS 2800 examination results are presented. Comparisons of sensors oriented to diesel fuel examination accessible in literature and described in this paper are presented in Table 3.

## 5. Conclusions

The proposed capillary sensor with disposable optrode of diesel fuel quality shows its ability to classify known and unknown fuel samples in a few minutes including one minute of sample examination in the sensor. The measured dynamic parameters of fuel vapor phase creation and presence proved to be crucial to fuel classification. The automatic outlier data elimination reduces classification errors to an acceptable minimum of less than 1/9. Initial digital signal filtering with proper data acquisition sampling time and use of five point’s first derivative calculation enables proper automatic conversion of data series to vector form required for classification. The optoelectronic circuit construction including initial signal filtering provides the possibility of fuel samples examination in an environment with saturated electromagnetic noise generated by WiFi networks. The local heating technology enables fast examination of fuel samples. The disposable capillary with marked points enables proper fuel sampling. All parts of the proposed hardware and software solutions are together essential for obtaining the presented results. 

The aim of the first set of planned future works is an extension of the test fuels set and carrying out extensive tests for the classification of fuels to which the sensor was not directly prepared. The next future works are aimed in the integration of sensor head with optoelectronics units. Thus, the fuel customer may have in the future a tool for correct and accurate assessment of diesel fuel quality.

## Figures and Tables

**Table 1:** Fuels type and condition versus basic petrochemical parameters of fuels used to sensor development.

Fuel Quality Type	Fuel Condition	CN	Min Density at 15 °C [kg/m^3^]	Kinematic Viscosity at 40 °C [mm^2^/s]		Fractional Composition of Distillation % [V/V]		Bio Component % [V/V]	Induction Time [h]
		min	min	min	max	up to 250 °C	up to 350 °C		
premium	good, fresh	55	820	2.0	4.5	65	85	0	20
standard	good, fresh	51	820	2.0	4.5	65	85	7	20
conditional acceptable	good, fresh	51	860	3.5	5.0	0	90	97	8
conditional acceptable	bad, out of date	characterized by sediment; not meets EU standards							

**Table 2:** Input vector processing for artificial neural networks classifier.

Vector Collected	Vector Passed for ANN Input
A0	-
Amin	Amin/A0
Amax	Amax/A0
Dmin	Dmin
Tdmin	Tdmin
Tdmax	Tdmax-Tdmin
Dmax	Dmax
A60	A60/A0

**Table 3:** Comparisons of sensors oriented to diesel fuel examination accessible in literature and described in this paper.

Sensor Type	Sensor Ref.	Sensing Parameters	Fuel under Analysis	Main Sensor Answer	Additional Sensor Answer
fluorescence sensor	[24]	time-resolved fluorescence with time of fluorescence decay	diesel, gasoline	fuel type: diesel, gasoline	gasoline type: E95, E98; diesel type: petro, bio
capillary sensor with UV–VIS reading	[25]	light scattering at UV, fluorescence emission at VIS	diesel	diesel fuel dated/outdated	pointing fuel storage over 2 years
capillary sensor with UV-forced degradation	[26]	fluorescence reading and UV degradation	diesel	diesel fuel stability	pointing fuel degradation
SCI fuel quality sensor and MEAS FPS 2800	[30]	viscosity, density, dielectric constant	liquid fuels	fuel type: petro-diesel, bio-diesel, gasoline, jet fuel	falsification of fuel, water pollution presence
dynamical capillary rise sensor	[31]	viscosity, density, surface tension	diesel	diesel fuel dated/outdated	pointing fuel storage over 2 years
fiber optic capillary sensor with smart optrode	[39]	initial distillation point, vapor pressure at distillation start, heat of evaporation	diesel	diesel fuel volume ratio of bio-component	falsification of fuel with edible oils
capillary sensor with local heating and data processing	This paper	initial distillation point, vapor pressure at distillation start, heat of evaporation surface tension, viscosity, heat of condensation	diesel	diesel fuel quality oriented to fuel user: premium fuel, standard fuel, acceptable fuel	pointing fuel storage over 3 years
